# Sustained chlorhexidine susceptibility in clinical MRSA isolates despite recent exposure

**DOI:** 10.1093/jacamr/dlag111

**Published:** 2026-06-16

**Authors:** Nadja Morello, Federica Andreoni, Willy I Staiger, Thomas C Scheier, David Weller, Ekaterina Vostokova, Silvio D Brugger

**Affiliations:** Department of Infectious Diseases and Hospital Epidemiology, University Hospital Zurich, University of Zurich, Zurich, Switzerland; Department of Infectious Diseases and Hospital Epidemiology, University Hospital Zurich, University of Zurich, Zurich, Switzerland; Department of Infectious Diseases and Hospital Epidemiology, University Hospital Zurich, University of Zurich, Zurich, Switzerland; Department of Infectious Diseases and Hospital Epidemiology, University Hospital Zurich, University of Zurich, Zurich, Switzerland; Department of Infectious Diseases and Hospital Epidemiology, University Hospital Zurich, University of Zurich, Zurich, Switzerland; Department of Infectious Diseases and Hospital Epidemiology, University Hospital Zurich, University of Zurich, Zurich, Switzerland; Department of Infectious Diseases and Hospital Epidemiology, University Hospital Zurich, University of Zurich, Zurich, Switzerland

## Abstract

**Background and objectives:**

MRSA decolonization strategies are widely implemented to reduce the risk of invasive infections but rely heavily on the use of chlorhexidine. Reduced chlorhexidine susceptibility has been reported after widespread use in hospital settings. The objective of the study is to assess chlorhexidine susceptibility among clinical MRSA isolates and to investigate potential induction of reduced susceptibility following chlorhexidine exposure.

**Methods:**

Ninety-five MRSA isolates from 77 patients of the University Hospital Zurich, treated between October 2021 and September 2022, were included. Chlorhexidine MICs were determined using the broth microdilution method, and subsequently MBCs were assessed. WGS was performed using the Illumina technology. Ten isolates with MBC values ≥8 mg/L were selected for cyclic exposure experiments using sub-inhibitory chlorhexidine concentrations and for analysis of genes associated with reduced chlorhexidine susceptibility. Longitudinal isolates from seven patients were analysed to assess in-host evolution.

**Results:**

Most isolates showed chlorhexidine MICs of 2 mg/L (76.6%, 60/77) and MBCs of 4 mg/L (68.8%, 53/77). ST8 was the most represented MLST among the isolates tested (23.1%). None of the 10 isolates with MBCs ≥8 mg/L demonstrated further reductions in chlorhexidine susceptibility following repeated sub-lethal exposure. Although resistance-associated genes were detected, no phenotypic impact on chlorhexidine susceptibility was observed. Longitudinal analysis of seven patients revealed stable MIC and MBC values despite prolonged carriage and chlorhexidine exposure.

**Conclusions:**

These findings indicate the absence of inducible or clinically relevant reduced susceptibility to chlorhexidine among MRSA isolates in this setting, despite repeated exposure.

## Introduction

*Staphylococcus aureus* is a human pathobiont that can colonize the host but also causes life-threatening infections. Invasive *S. aureus* infections are often difficult to treat and linked to high morbidity and mortality. The emergence of MRSA further complicates treatment, due to the inefficacy of first-line antibiotic therapy. Colonization with MRSA has been recognized as a risk factor for subsequent infection, and decolonization protocols aim to eradicate MRSA and therefore lower this risk.^1–3^ Decolonization is carried out using antiseptic (e.g. chlorhexidine mouthwashes and washing lotions, and nose creams containing mupirocin).^4,5^ Chlorhexidine is a bis-biguanide used as an antiseptic agent since the 1970s, owing to its broad-spectrum activity against bacteria, fungi and viruses.^6,7^ Chlorhexidine is widely employed as a gold-standard antiseptic in dentistry and oral care, as a skin antiseptic preceding surgical procedures, and for the disinfection of surfaces and medical devices.^8^

Reduced susceptibility of *S. aureus*, including MRSA, towards commonly used decolonization agents is a major concern since increased usage of chlorhexidine has been associated with lower susceptibility, thereby reducing the effectiveness of decolonization procedures.^9,10^ Decreased chlorhexidine susceptibility has been associated with the presence of multidrug efflux pump resistance genes specific to the transport of quaternary ammonium compounds (QACs), belonging to the small multidrug resistance (SMR) family.^11^ An increase in efflux pump activity, driven by acquisition of plasmid-borne multidrug efflux pumps or by overexpression of multidrug efflux pump genes, is a common mechanism leading to decreased chlorhexidine susceptibility.^7^

Exposure to sub-lethal concentrations of chlorhexidine has been shown to lead not only to reduced susceptibility towards this compound but also to cross-resistance to other anti-infective agents.^12^ Besides reducing decolonization effectiveness in patients, decreased susceptibility to chlorhexidine would also jeopardize widely implemented infection prevention procedures, such as its use for washing in intensive care units (ICUs) or as preoperative antiseptic.^13^ Despite these concerns, the prevalence of reduced susceptibility to chlorhexidine in MRSA remains under-monitored. This study investigated the prevalence of reduced chlorhexidine susceptibility, its possible induction and the presence of chlorhexidine resistance–associated genes in MRSA clinical isolates.

## Material and methods

### Bacterial strains

Ninety-five MRSA strains, isolated from 77 patients at the University Hospital Zurich (USZ) between October 2021 and September 2022, were included in this study (Tables 1 and 2). The sample size was dictated by the number of MRSA isolates that were recovered in this time frame. Outpatients’ swabs were collected in the outpatient clinic of the Department of Infectious Diseases and Hospital Epidemiology of USZ, while inpatients’ swabs were collected from various wards across the USZ, lowering the chance of epidemiological links among the isolates. For the assessment of chlorhexidine decreased susceptibility prevalence, only one isolate per patient was assessed (Table 1). For patients P01, P03, P04, P06, P07, P09 and P14, multiple isolates were analysed to test for inpatient evolution of MRSA in the presence or absence of exposure to chlorhexidine (Table 2). The isolates were cultured overnight at 37°C on Columbia agar + 5% sheep blood plates (COS, bioMérieux SA).

**Table 1.** Clinical isolates and patients characteristics

	CHX exposure									Patient				First finding	
Patient CHX exposure 28 d^a^	Reason^a^	Duration,^a^ d	MBC,^b^ mg/L	MIC,^b^ mg/L	CHX exposure challenge^c^	CHX resistance genes^d^	Isolate ID	MLST	Isolation source^e^	Sex	Age	Inpatient/outpatient	Screening/symptoms	Patient^f^	365 days^g^
No	NA	NA	2	2	No	ND	P58-1	ND	Deep wound	f	67	inpatient	**symptoms**	no	no
							P15-1		Inguinal	m	29	inpatient	screening	no	no
							P72-1			m	19	outpatient	screening	no	no
							P17-1		Nasopharyngeal	m	56	inpatient	screening	no	no
							P30-1			m	65	inpatient	screening	no	yes
							P80-1			m	53	outpatient	screening	yes	yes
							P65-1		Nose	m	47	outpatient	screening	yes	yes
							P79-1			m	74	inpatient	screening	no	no
							P25-1		Throat	f	21	outpatient	screening	yes	yes
							P48-1			f	51	outpatient	screening	no	no
							P52-1			m	27	inpatient	screening	no	no
							P62-1		Umbilical/perineal	m	0	inpatient	screening	yes	yes
							P81-1			f	0	outpatient	screening	no	no
			4	1	No	ND	P64-1	ND	Ear	m	77	inpatient	screening	no	no
							P46-1		Inguinal	m	34	outpatient	screening	no	no
							P28-1		Nasopharyngeal	m	69	inpatient	screening	no	no
							P32-1			m	59	inpatient	screening	no	yes
							P34-1			f	35	inpatient	screening	no	no
							P51-1			m	78	inpatient	screening	no	no
							P54-1			f	47	inpatient	screening	no	yes
							P60-1			f	87	inpatient	screening	no	no
							P66-1			m	66	inpatient	screening	no	yes
							P21-1		Nose	m	58	outpatient	screening	yes	yes
							P22-1		Superficial wound	m	77	inpatient	**symptoms**	no	yes
							P31-1			f	71	inpatient	**symptoms**	no	no
							P43-1		Throat	f	12	inpatient	screening	no	no
							P49-1		Deep wound	m	41	inpatient	**symptoms**	nof	no
				2	No	ND	P19-1	ND	Inguinal	m	85	inpatient	screening	no	yes
							P59-1			m	41	outpatient	screening	yes	yes
							P69-1			m	60	inpatient	screening	yes	yes
							P16-1		Nasopharyngeal	m	0	inpatient	screening	yes	yes
							P20-1			f	42	outpatient	screening	no	no
							P37-1			m	48	inpatient	screening	no	no
							P38-1			m	82	inpatient	screening	no	no
							P40-1			m	35	outpatient	screening	yes	yes
							P41-1			m	88	inpatient	screening	yes	yes
							P44-1			m	35	inpatient	screening	yes	yes
							P45-1			f	46	inpatient	screening	no	yes
							P47-1			f	41	inpatient	screening	no	no
							P53-1			f	63	inpatient	screening	no	yes
							P57-1			m	0	inpatient	screening	yes	yes
							P61-1			f	26	inpatient	screening	no	no
							P71-1			f	43	inpatient	screening	no	yes
							P73-1			m	57	inpatient	screening	no	no
							P75-1			f	22	outpatient	screening	no	no
							P76-1			f	0	inpatient	screening	yes	yes
						*norAI*	**P04-1**	1	Nose	f	21	outpatient	screening	yes	yes
						ND	P24-1	ND		m	55	inpatient	screening	no	yes
							P29-1			f	31	inpatient	screening	no	no
							P50-1			m	39	inpatient	screening	no	yes
							P56-1			m	22	inpatient	screening	yes	yes
							P74-1			m	57	inpatient	screening	no	no
							P78-1		Rectal	m	42	inpatient	screening^a^	no	no
							P36-1		Superficial wound	m	65	inpatient	**symptoms**	yes	yes
							P33-1		Throat	f	24	outpatient	screening	no	no
							P68-1		Vaginal	f	49	outpatient	screening^h^	yes	yes
							P70-1			f	40	inpatient	**symptoms**	yes	yes
				4	No	ND	P35-1	ND	Nasopharyngeal	m	77	inpatient	screening	no	no
			8	2	Yes	*norAI, qacC*	P02-1	8	Nasopharyngeal	f	40	inpatient	screening	no	yes
						*norAI*	P12-1	5050		f	19	outpatient	screening	no	no
						*norAI*	P08-1	8	Superficial wound	f	28	inpatient	**symptoms**	no	no
			16	1	Yes	*norAI*	**P03-1**	5	Inguinal	m	20	inpatient	screening	no	no
				2	Yes	*norAII*	P13-1	30	Nasopharyngeal	f	55	inpatient	screening	no	no
						*norAII*	**P09-1**	22	Nose	m	43	inpatient	screening	no	no
						*norA*	P10-1	59		m	75	inpatient	screening	no	no
						*norAII*	P11-1	22		m	64	inpatient	screening	no	no
						*norAI*	**P07-1**	5	Superficial wound	m	63	inpatient	**symptoms**	no	no
Yes	Decolonization	5	2	2	No	ND	P63-1	ND	Inguinal	f	40	outpatient	screening	yes	yes
	Decolonization	10	4	1	No	ND	P18-1		Throat	f	36	outpatient	screening	no	no
	Decolonization	5					P39-1			f	29	inpatient	screening	no	no
	Decolonization	10		2	No	ND	P26-1	ND	Nasopharyngeal	m	33	outpatient	screening	yes	yes
	ICU	3					P42-1			f	88	inpatient	screening	no	no
	Decolonization	10				*norAI*	**P01-1**	10 931	Throat	f	31	outpatient	screening	no	no
	Decolonization	5				*norAI*	**P14-1**	8		f	28	outpatient	screening	no	no
	Decolonization	7				ND	P23-1	ND		m	57	outpatient	screening	no	no
	Decolonization	10					P55-1			m	32	outpatient	screening	no	no
	Decolonization	10	16	2	Yes	*norAI*	**P06-1**	10 931	Throat	m	32	inpatient	screening	no	no

NA, not available; ND, not determined.

Seventy-seven swabs were taken from the same number of inpatients or outpatients of the University Hospital Zurich between October 2021 and September 2022. For seven patients (P01, P03, P04, P06, P07, P09 and P14), multiple isolates were collected and sequenced (Table 2). The first isolates stemming from these patients are also included in this table and indicated in bold.

^a^Ten of 77 patients were exposed to chlorhexidine (CHX) in the 28 days preceding the collection of the swab. The reasons for exposure, either decolonization procedures or stay in the ICU, are also stated, as well as the duration of the exposure to chlorhexidine.

^b^Chlorhexidine MBCs and MICs were measured by broth microdilution assay in Mueller–Hinton broth.

^c^The 10 isolates demonstrating an MBC of 8 mg/L or higher were subject to eight consecutive cycles of chlorhexidine exposure.

^d^The MLST and the presence of genes coding for multidrug efflux pumps and associated with chlorhexidine decreased susceptibility were determined in a subset of strains.

^e^The 77 swabs originated from superficial or deep body sites and were taken for screening purposes or from patients presenting with symptoms.

^f^The isolates stemmed either from the first known MRSA-positive swab in a patient or from patients who had previously had positive swabs.

^g^Column indicates whether the swab was the first positive swab within 365 days.

^h^Screening for additional reservoir sites following unsuccessful decolonization procedure.

**Table 2.** Clinical isolates tested for inpatient evolution

Patient ID	Isolate ID^a^	Isolation day compared with first isolate	Patient CHX exposure 28 days^b^	MIC,^c^ mg/L	MBC,^c^ mg/L	MLST^d^	*norA* type^d^
P01	**P01-1**	0	Yes	2	4	10 931	*norAI*
	P01-2	29	Yes	2	16	10 931	*norAI*
	P01-3	62	Yes	1	4	10 931	*norAI*
P03	**P03-1**	0	No	1	16	5	*norAI*
	P03-2	123	No	2	2	5	*norAI*
	P03-3	201	No	2	2	5	*norAI*
	P03-4	326	No	2	2	5	*norAI*
P04	**P04-1**	0	No	2	4	1	*norAI*
	P04-2	14	Yes	1	4	1	*norAI*
	P04-3	35	No	1	4	1	*norAI*
	P04-4	73	Yes	2	4	1	*norAI*
	P04-5	181	Yes	2	8	1	*norAI*
P06	**P06-1**	0	Yes	2	16	10 931	*norAI*
	P06-2	33	Yes	1	2	10 931	*norAI*
	P06-3	285	No	2	8	10 931	*norAI*
P07	**P07-1**	0	No	2	16	5	*norAI*
	P07-2	96	No	2	4	5	*norAI*
	P07-3	179	No	4	8	5	*norAI*
	P07-4	181	No	4	4	5	*norAI*
P09	**P09-1**	0	No	2	16	22	*norAII*
	P09-2	57	Yes	2	2	22	*norAII*
	P09-3	262	No	2	4	22	*norAII*
P14	**P14-1**	0	Yes	2	4	8	*norAI*
	P14-2	17	Yes	2	4	8	*norAI*
	P14-3	63	Yes	2	2	8	*norAI*

Isolates indicated in bold are also included in Table 1.

^a^Multiple isolates were samples from patients P01, P03, P04, P06, P07, P09 and P14.

^b^The isolation day, compared with the first isolate used in this study (set to ‘Day 0’), as well as whether a patient was exposed to chlorhexidine (CHX) in the 28 days preceding isolation are indicated. The isolation date of the first isolates included in this study did not necessarily match with recent (<28 days) exposure to chlorhexidine in the patient.

^c^Chlorhexidine MIC and MBC assessment as well as WGS were carried out on the strains to follow inpatient evolution of susceptibility to chlorhexidine.

^d^MLST and *norA* gene STs were also determined to follow inpatient evolution of susceptibility to chlorhexidine.

### MIC and MBC assessment

Chlorhexidine digluconate (Sigma Aldrich) was used to determine MICs and MBCs. Chlorhexidine MICs were measured by broth microdilution. Bacteria were cultured overnight at 37°C on Columbia agar + 5% sheep blood (COS) plates from stocks conserved at −80°C. Bacteria were subsequently inoculated in CAMBH II (Becton Dickinson) and grown for 16 h at 37°C shaking. Overnight cultures were diluted in CAMBH to a concentration of 5 × 10^5^ cfu/mL, exposed to chlorhexidine concentrations ranging from 0.03125 to 16 mg/L (1:2 dilution steps of the drug) and incubated for 18–22 h at 37°C. The breakpoint for decreased susceptibility to chlorhexidine was 8 mg/L.^14^ MBCs were determined for all clinical isolates by plating 10 µL of culture from each well containing a chlorhexidine concentration that inhibited bacterial growth in the MIC assay on tryptic soy broth plates (TSB; Becton Dickinson), followed by incubation for 24 h at 37°C.^15^ The MBC was defined as the lowest chlorhexidine concentration where no growth on plates was observed.

### Cyclic exposure to chlorhexidine sub-lethal concentrations

Isolates demonstrating a chlorhexidine MBC ≥8 mg/L, suggested as a breakpoint MIC for *in vitro* studies,^14^ were included in this experiment. We inoculated 100 µL of the bacterial suspension demonstrating growth at the highest chlorhexidine concentration (1 dilution below the MIC) in the MIC assay in 900 µL CAMBH, and the optical density at 600 nm (OD_600_) of these cultures was assessed. The bacterial concentration was subsequently adjusted to 5 × 10^5^ cfu/mL, by further diluting the cultures, based on the OD_600_ values. The OD_600_ values corresponding to the correct inoculum for the MIC/MBC assessment were determined in preliminary assessments by plating serial dilution of the cultures. Chlorhexidine MICs and MBCs were reassessed using chlorhexidine concentrations ranging from 0.125 to 64 mg/L (1:2 dilution steps of the drug). After incubation at 37°C for 18–22 h, the MICs and MBCs were determined, and the same process was repeated daily. Eight cycles of chlorhexidine exposure followed by MIC and MBC assessment were carried out over the following eight days.^16^ Since only up to 4-fold MIC or MBC fluctuations were observed, rather than consistent or permanent changes, the experiment was terminated after 8 days. Bacteria recovered from wells demonstrating growth at the highest chlorhexidine concentration were diluted at least 400 times before MIC and MBC determination, which ensured no carryover of relevant amounts of chlorhexidine to the new assay.

### WGS

The 10 strains with an MBC ≥8 mg/L were sequenced to test for the presence of proteins associated with reduced chlorhexidine susceptibility, the sequences of which are listed in Table S1 (available as Supplementary data at *JAC-AMR* Online). WGS was also carried out on multiple strains isolated at different timepoints from the same patient, to follow inpatient evolution of the parental strain. All strains were sequenced at the Institute of Medical Microbiology of the University of Zurich using the Illumina technology, as previously described.^17^ Assemblies were screened for the presence of corresponding proteins using the ‘mmseqs search’ command with 70% identity threshold, using the reference sequences summarized in the Supplementary material (Table S1). Potential hits were checked with protein BLAST.^18,19^ To estimate the number of core genome SNPs, snp-dists 0.8.2 was run on the core genome alignment across all first isolates from P1–P4 and P6–P14 (Table S2). The sequences were deposited to the European Nucleotide Archive (ENA, project number SUB15022020).

## Results

### Patients’ characteristics

The patients’ median age was 42 years (range 0–88 years), 32 were female (42%), 54 were inpatients (70%) and 23 outpatients (30%). (Table 1). The 77 isolates originated from superficial (91%, 70/77) or deep (9%, 7/77) body sites. In 21 patients (27%) the respective isolate was the first known MRSA-positive swab in said patient. Ten of the 77 patients included in this study were exposed to chlorhexidine for a maximum of 28 days prior to the collection of the swab, either for decolonization procedures (90%) or as part of routine body washes during treatment in the ICU (10%) (Table 1).

### Absence of decreased susceptibility to chlorhexidine

**Figure 1.**
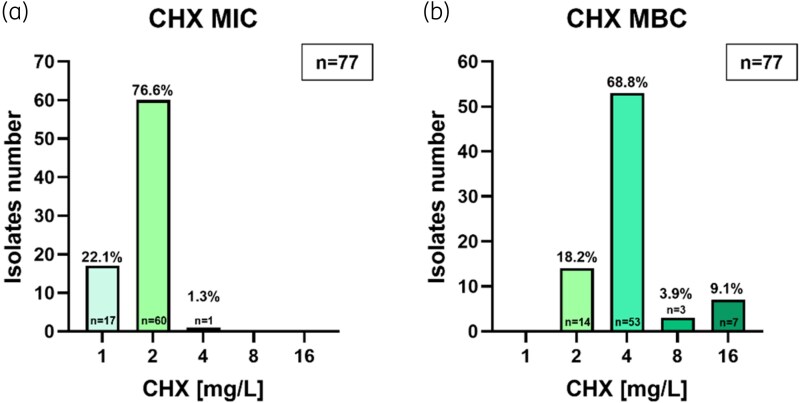
(a) Chlorhexidine (CHX) MIC and (b) MBC distribution of the 77 MRSA strains tested. The relative percentages and number of strains displaying the indicated MIC or MBC can be found on each bar; the total number of isolates is indicated in the boxes.

For the assessment of chlorhexidine decreased susceptibility prevalence, only one isolate per patient was included in this analysis (Table 1). None of the strains showed a chlorhexidine MIC ≥8 mg/L (Figure 1a), indicating the absence of reduced susceptibility towards the drug. Sixty strains out of 77 (76.6%) displayed an MIC of 2 mg/L. MBCs spanned between 2 and 16 mg/L, with 53 strains (68.8%) displaying an MBC of 4 mg/L (Figure 1b). MIC and MBC distributions were not associated with previous exposure of patients to chlorhexidine (Figure S1).

### Chlorhexidine resistance evolution experiment

**Figure 2.**
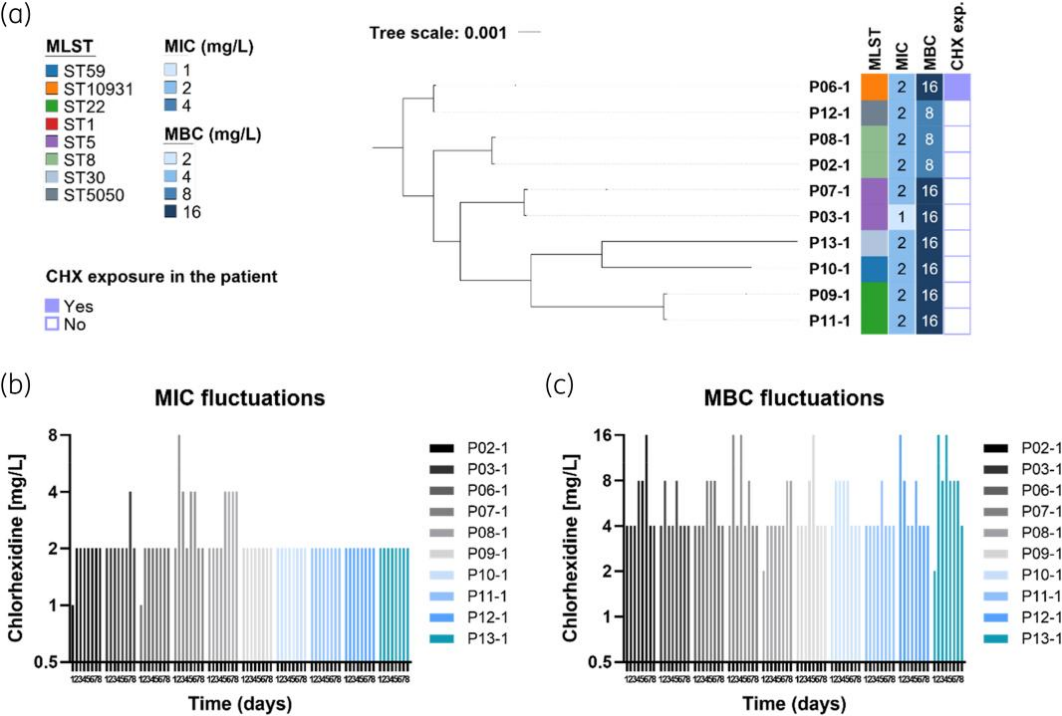
Baseline characteristics of the 10 isolates demonstrating a chlorhexidine MBC ≥8 mg/L and chlorhexidine exposure challenge. (a) Core genome–based maximum-likelihood tree, highlighting MLST, MIC, MBC and whether the patient was exposed to chlorhexidine (CHX) 28 days prior to isolation of the strains (CHX exposure in the patient). The scale bar indicates the number of nucleotide substitutions per site. (b) Chlorhexidine MIC and (c) MBC fluctuations upon continuous exposure to chlorhexidine carried out on the 10 strains demonstrating an MBC ≥8 mg/L. Eight 24 h cycles of chlorhexidine exposure followed by MIC and MBC assessment were carried out.

The 10 isolates demonstrating an MBC ≥8 mg/L (Table 1) were selected to assess whether continuous exposure to sub-lethal chlorhexidine concentrations *in vitro* would lead to decreased susceptibility to the drug in these isolates. The baseline characteristics of these isolates were first assessed. WGS was carried out to determine their MLST and to assess the presence of genes typically associated with decreased chlorhexidine susceptibility (Table S1). WGS revealed the presence of seven different MLSTs, including a novel one, ST10391 (Figure 2a). The MLST did not correlate with chlorhexidine susceptibility in this cohort. The presence of *norA*, associated with decreased chlorhexidine susceptibility, was detected in all strains, and the *qacC* was present only in one strain (Table S1). Upon continuous exposure to chlorhexidine, MIC and MBC fluctuations of up to 4-fold were observed in some of the strains, but no induction of a permanent increase in MICs or MBCs was detected over the 8 days of exposure (Figure 2b and c).

### Inpatient isolates evolution

**Figure 3.**
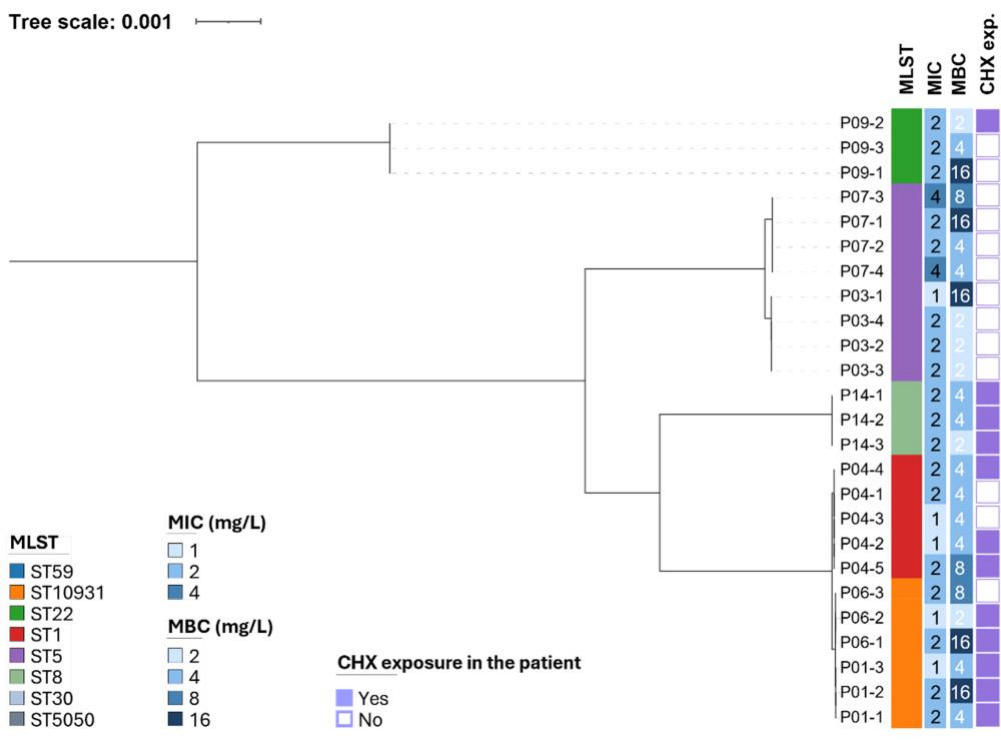
Inpatient isolates evolution. Core genome–based maximum likelihood tree of isolates sampled at different timepoints from the same seven patients, indicating MLST, chlorhexidine (CHX) MIC and MBC as well as chlorhexidine exposure in the patient in the 28 days preceding isolation. The scale bar indicates the number of nucleotide substitutions per site.

To assess whether exposure of the patients to chlorhexidine as well as persistence in the host over a prolonged period would affect chlorhexidine MICs and MBCs of isolates as well as their genetic traits, we selected seven patients for which multiple MRSA isolates were collected (Table S2). The times of isolation spanned between 14 and 326 days from the first isolate sampled in each patient. MICs and MBCs fluctuated slightly among isolates of the same patient (Table S2), but we did not find a correlation between MIC/MBC values and chlorhexidine exposure in the patient or persistence in the host (Figure 3).

A core genome–based tree showed that the strains colonizing each patient remained the same, differing only by a limited number of SNPs. The SNPs were different for each patient, with no detectable patterns of genes involved (Figure 3 and Table S3). WGS analysis also showed that the *norA* gene sequence did not accumulate mutations among isolates longitudinally collected from the same patient (Figure S2a).

### MLST and NorA variant distribution

MLST as well as the sequence of the *norA* gene were analysed in the 13 isolates used for inpatient evolution studies, including the 10 isolates that showed MBCs ≥8 mg/L (Table 1). Only the first isolate sampled from each patient was considered in this analysis. ST8 was the most prevalent MLST (3/13 strains, 23%); a new MLST (ST10931) was identified for two isolates (Figure S2b). Sequence analysis of the NorA protein revealed the presence of WT NorA (1/13 strains, 8%) as well as of the NorAI (9/13 strains, 69%) and NorAII (3/13 strains, 23%) variants (Figure S2c), previously described in the literature.^20^ Several isolates carried mutations in NorAI (P07: Gly291Asp; P02, P08 and P14: Gly147Ser)^20–22^ and NorAII (P09 and P11: Asn200Asp)^21^ (Figure S2a). The QacC protein, found in one isolate, did not display functional mutations compared with the WT sequence. We additionally determined the number of SNPs present in the core genome of these isolates and detected a maximum distance of 29 731 SNPs between isolates P01-1 and P13-1 and a minimum distance of 10 SNPs between isolates P08-1 and P14-1.

## Discussion

In this study, we assessed decreased chlorhexidine susceptibility prevalence in 77 MRSA isolates coming from different patients and investigated the presence of genes associated with reduced susceptibility to chlorhexidine as well as changes in chlorhexidine susceptibility in strains undergoing cyclic exposure to chlorhexidine. We found no decreased chlorhexidine susceptibility in this cohort nor a correlation between MLST and chlorhexidine MICs/MBCs, but we could detect the presence of the *norA* gene in all isolates and of *qacC* in one isolate. Predominantly, ST8, ST5 and ST22 isolates were found, as previously reported for Switzerland.^23^ Cyclic exposure of the MRSA isolates to sub-lethal doses of chlorhexidine did not lead to the development of decreased susceptibility to the compound, and no mutations were accumulated in isolates repeatedly sampled from the same patient, also in patients exposed to chlorhexidine.

Decreased susceptibility to chlorhexidine has been described in clinical settings including ICUs, and resistance surveillance was deemed necessary by several authors,^24–26^ although prolonged exposure to chlorhexidine in the host did not always lead to decreased susceptibility.^26^ Chlorhexidine MICs and MBCs of the MRSA isolates included in this work are in line with other studies investigating *S. aureus*, showing mostly chlorhexidine MICs of 2 and 4 mg/L.^14,27^ These studies were also the foundation for the epidemiological cut-off values (ECOFFs) for chlorhexidine MIC (≥8 mg/L) and MBC (>64 mg/L). According to this definition, all 95 isolates screened in this work displayed WT susceptibility to chlorhexidine.

The 10 isolates displaying an MBC ≥8 mg/L were further selected for a decreased susceptibility-induction experiment, carried out by exposing the isolates to sub-inhibitory concentrations of chlorhexidine for 8 days. Despite a chlorhexidine exposure longer than the recommended course of chlorhexidine application in decolonization protocols,^28^ no reduction of susceptibility was detected in the 10 isolates screened in this study.

Both findings reassure that chlorhexidine remains a valid option for decolonization protocols. However, these results are in contrast with previous reports suggesting that sub-lethal concentrations of chlorhexidine can induce reduction of susceptibility in MRSA. One study assessed the MICs and MBCs of *S. aureus* strains isolated before and after the use of chlorhexidine was implemented in hospital settings in the UK, showing a steady increase in both across one century (1920 to early 2000s) although isolates displaying decreased susceptibility to chlorhexidine (MIC ≥ 8 mg/L) were already present before the use of chlorhexidine was introduced.^9^ The average MIC values for the chlorhexidine era were, however, between 4 mg/L and 64 mg/L, higher values than those we observed. In a review by Kampf,^10^ the reaction of many bacterial species to chlorhexidine exposure was described. For *S. aureus*, an MIC increase of 0- to 16-fold was observed upon exposure to sub-lethal concentrations of chlorhexidine, but in most cases the stability of the acquisition of an increased MIC was either unknown or not present. Particular attention should be paid to chlorhexidine exposure for Gram-negative bacteria such as *Escherichia coli*, where much higher (up to 500-fold) MIC increases have been reported. Chlorhexidine exposure has been associated with the activation of horizontal gene transfer, causing an increase in the spread of antibiotic resistance.^10^ In our case, during the resistance-induction experiment, we observed only up to 4-fold MIC fluctuations (between 2 and 8 mg/L) in 1 out of 10 strains, and MBC fluctuation between 4 and 16 mg/L in 4 out of the 10 strains tested. The sample size we utilized in this work, and the regionality of the isolates might bias this result. While the lack of MIC increase is reassuring, a temporary increase in MBCs might indicate persistence of a subset of cells that could lead to incomplete eradication and later recurrence of MRSA colonization, even if formal requirements for the definition of tolerance are not met (MBC/MIC ratio >32).^29^

Several quaternary ammonium compound (*qac*) resistance genes, as well as *norA*, coding for efflux pumps, are involved in decreased susceptibility to chlorhexidine.^11^ None of the *qac* genes, apart from the presence of *qacC* in one isolate, were found in the genomes of the isolates tested in this work, while *norA* was present in all isolates. The lack of decreased phenotypic susceptibility towards chlorhexidine in our cohort questions the clinical importance of the presence of the multidrug efflux pump resistance genes *norA* and *qacC*, linked to cross-resistance to chlorhexidine and antibiotics.^25,30^ Our findings contrast with previous studies, where an association between these resistance genes and chlorhexidine susceptibility was suggested,^24,31^ but are in line with existing literature showing no effect of *norA* or *qacC* presence on chlorhexidine MICs in MRSA isolates.^32^ Efflux pump–related resistance is moreover attributable not only to the presence of the genes but also to their expression levels.^7^ In addition, *norA* has been shown to be part of the core genome of *S. aureus* and most streptococcal species,^21^ suggesting caution while establishing a correlation between its presence and decreased chlorhexidine susceptibility.^20,21^ The assessment of expression levels in phenotypically less susceptible isolates, as well as the presence and integrity of such genes, should be implemented in the future to investigate whether expression levels of a functional protein strictly correlate with decreased chlorhexidine susceptibility.

A closer look at the sequence of the *norA* gene revealed the presence of NorAI and NorAII alleles, as well as the WT NorA. The NorAI allele was the most represented in the isolates analysed, as previously reported,^20^ and was mainly associated with ST1, ST5 and ST8, while the NorAII allele was associated with ST59 and ST22. None of the strains carried the NorAIII variant, commonly associated with ST45.^20^ Many of the mutations found in the NorA allele variants fall in transmembrane segment regions and do not seem to affect the function of the efflux pump,^20^ as also suggested by our results. The mutation Gly147Ser, located in a conserved motif of the transmembrane region 5 involved in substrate binding in NorAI,^20^ was found in three isolates and is predicted to affect the function of the efflux pump. However, we observed no increase in susceptibility to chlorhexidine in these isolates. The other mutation found in NorAI (Gly291Asp) was previously described and not deemed to influence the functionality of the efflux pump.^21,22^ Finally, the mutation Asn200Asp was also previously described in the literature in ST22 strains,^21^ as reported in this work. We can therefore conclude that the mechanisms by which MRSA acquires decreased susceptibility to chlorhexidine might not solely rely on the presence of a functional NorA transporter but probably depend on other factors.

In conclusion, decreased phenotypic susceptibility to chlorhexidine, defined as an MIC ≥ 8 mg/L, was not observed in the 77 isolates tested in this work. Moreover, reduced susceptibility could not be induced by repeated exposure to sub-lethal concentrations of chlorhexidine, despite the presence of resistance-associated genes. These findings suggest that chlorhexidine decolonization protocols for MRSA do not necessarily result in enhanced decreased susceptibility to chlorhexidine.

## Supplementary Material

dlag111_Supplementary_Data

## Supplementary data

Figures S1 and S2 and Tables S1–S3 are available as Supplementary data at *JAC-AMR* Online.
