# Supplementary material for: Sustained chlorhexidine susceptibility in clinical MRSA isolates despite recent exposure
Source: JAC Antimicrob Resist. 2026 Jun 16;8(3):dlag111. doi: 10.1093/jacamr/dlag111 (PMC13270236; doi:10.1093/jacamr/dlag111)
Supplement: dlag111_Supplementary_Data [file dlag111_supplementary_data.docx]

**Figure S1**

**
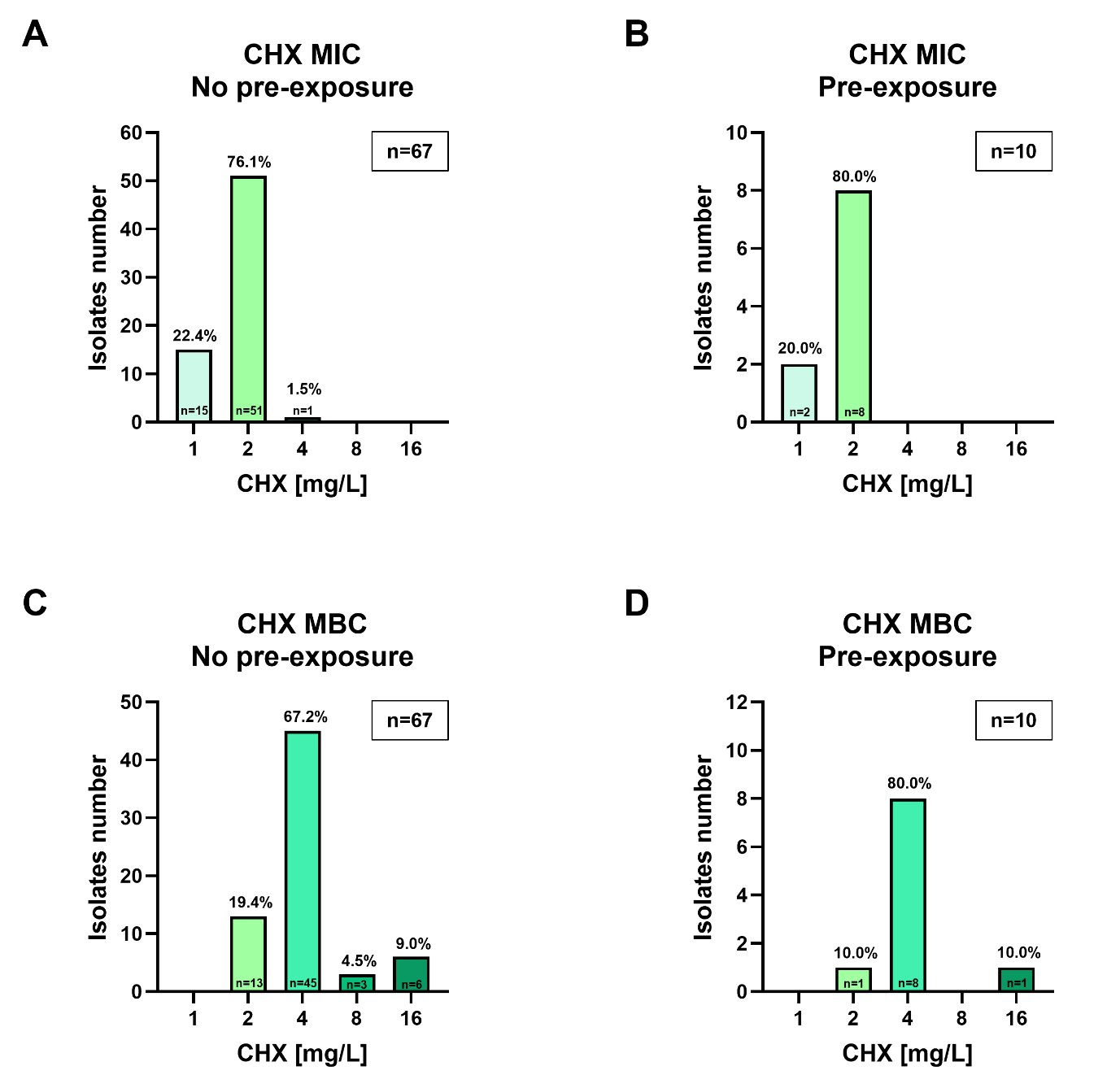
**

**Figure S1 - Minimal inhibitory and minimal bactericidal concentrations are not influenced by patients’ pre-exposure to chlorhexidine**

Minimal inhibitory concentration (MIC) and minimal bactericidal concentration (MBC) of clinical isolates either not pre-exposed (**A** and **C**) or pre-exposed (**B** and **D**) to chlorhexidine (CHX) in the patient in the 28 days preceding isolation. The percentage (**%**) and number of strains (**n=**) corresponding to the various MIC and MBC values are indicated on each bar of the graphs. The total number of strains tested is indicated in the box on each graph.

**Figure S2**

**
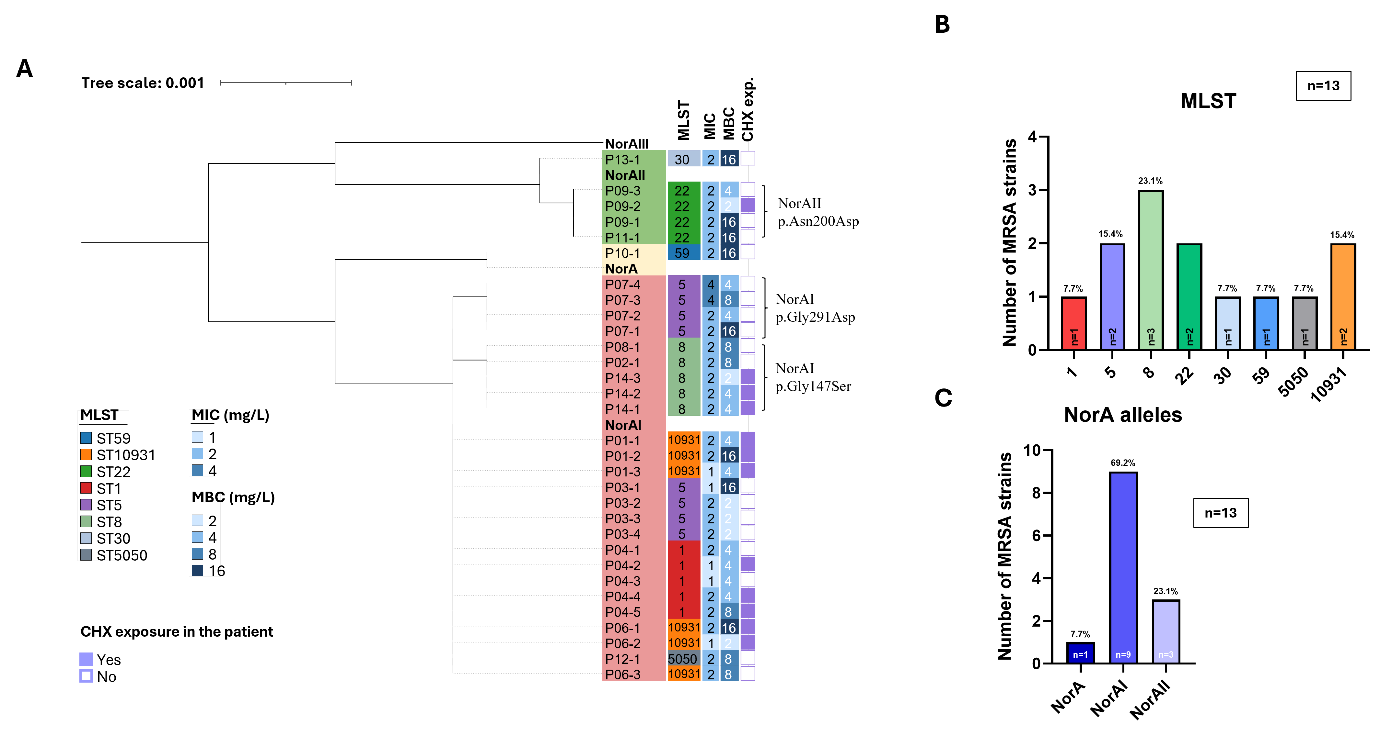
**

**Figure S2 - Multilocus sequence type and NorA variants distribution**

**A**) Maximum-likelihood tree based on the sequence of the Nora protein including all isolates sequenced in this work. The following sequences of the NorA protein and its variants were retrieved for the alignment: NorA-CC59/121 AII55214.1, NorAI sp/P0A0J7/NORA STAAU, NorAII tr/Q9ZNA9/ Q9ZNA9STAAU, NorAIII tr/Q03325/Q03325 STAAU ^34^. Multilocus sequence type (MLST), chlorhexidine (CHX) minimal inhibitory concentration (MIC) and minimal bactericidal concentration (MBC) are also displayed on the tree, as well as exposure of the patient to chlorhexidine in the 28 preceding isolation. On the right end side, variants of the NorAI and NorAII sequences are indicated. The scale bar indicates number of nucleotide substitutions per site. **B**) MLST distribution of 13 isolates. **C**) NorA alleles distribution of 13 isolates.

**Table S1**

| **Product name** | **Function** | **Genbank / NCBI ID** | **Length (aminoacids)** |
| --- | --- | --- | --- |
| QacA | Multidrug efflux pump | [ADK23699.1](https://www.ncbi.nlm.nih.gov/protein/300492224) | 514 |
| QacB | Multidrug efflux pump | [AAC38784.1](https://www.ncbi.nlm.nih.gov/protein/3327948) | 514 |
| QacC=QacD=smr | Multidrug efflux pump | [BAP94573.1](https://www.ncbi.nlm.nih.gov/protein/BAP94573.1) | 107 |
| QacE | Multidrug efflux pump | [QFK45777.1](https://www.ncbi.nlm.nih.gov/protein/QFK45777.1) | 102 |
| QacG | Multidrug efflux pump | [WP_251356105.1](https://www.ncbi.nlm.nih.gov/protein/WP_251356105.1) | 107 |
| QacH/QacI | Multidrug efflux pump | [CAA76544.1](https://www.ncbi.nlm.nih.gov/protein/CAA76544.1) | 107 |
| QacJ | Multidrug efflux pump | [CAD55144.1](https://www.ncbi.nlm.nih.gov/protein/CAD55144.1) | 107 |
| NorA | Multidrug efflux pump | [BAA14147.1](https://www.ncbi.nlm.nih.gov/protein/216975) | 388 |

**Table S1 - Sequences of *S. aureus* proteins involved in chlorhexidine resistance used to search for chlorhexidine resistance genes in the MRSA clinical isolates.**

The proteins listed in column one are involved in promoting chlorhexidine reduced susceptibility in S. aureus based on the literature. In column three the published sequence used to search for these proteins in the ten strains analyzed in this work is provided as a web-link. The sequence of QacC was confirmed with http://bacmet.biomedicine.gu.se.

**Table S2**

|  | **P01-1^10931^** | **P10-1^59^** | **P11-1^22^** | **P12-1^5050^** | **P13-1^30^** | **P14-1^8^** | **P02-1^8^** | **P03-1^5^** | **P04-1^1^** | **P06-1^10931^** | **P07-1^5^** | **P08-1^8^** | **P09-1^22^** |
| --- | --- | --- | --- | --- | --- | --- | --- | --- | --- | --- | --- | --- | --- |
| **P01-1^10931^** | 0 | 25558 | 20829 | 172 | **29731** | 10075 | 10089 | 11683 | 136 | 50 | 11575 | 9983 | 20817 |
| **P10-1^59^** | 25558 | 0 | 27167 | 24368 | 27072 | 25547 | 25573 | 25648 | 25532 | 25515 | 25445 | 25351 | 27336 |
| **P11-1^22^** | 20829 | 27167 | 0 | 20407 | 27277 | 21201 | 21183 | 21122 | 20810 | 20819 | 21014 | 20780 | 367 |
| **P12-1^5050^** | 172 | 24368 | 20407 | 0 | 29356 | 9995 | 10008 | 11425 | 155 | 168 | 11330 | 9905 | 20403 |
| **P13-1^30^** | **29731** | 27072 | 27277 | 29356 | 0 | 29552 | 29582 | 29592 | 29729 | 29728 | 29512 | 29063 | 27193 |
| **P14-1^8^** | 10075 | 25547 | 21201 | 9995 | 29552 | 0 | 540 | 11680 | 10068 | 10093 | 11642 | **10** | 21232 |
| **P02-1^8^** | 10089 | 25573 | 21183 | 10008 | 29582 | 540 | 0 | 11693 | 10083 | 10111 | 11662 | 531 | 21219 |
| **P03-1^5^** | 11683 | 25648 | 21122 | 11425 | 29592 | 11680 | 11693 | 0 | 11676 | 11664 | 393 | 11595 | 21154 |
| **P04-1^1^** | 136 | 25532 | 20810 | 155 | 29729 | 10068 | 10083 | 11676 | 0 | 175 | 11568 | 9977 | 20800 |
| **P06-1^10931^** | 50 | 25515 | 20819 | 168 | 29728 | 10093 | 10111 | 11664 | 175 | 0 | 11552 | 10001 | 20809 |
| **P07-1^5^** | 11575 | 25445 | 21014 | 11330 | 29512 | 11642 | 11662 | 393 | 11568 | 11552 | 0 | 11557 | 21047 |
| **P08-1^8^** | 9983 | 25351 | 20780 | 9905 | 29063 | **10** | 531 | 11595 | 9977 | 10001 | 11557 | 0 | 20817 |
| **P09-1^22^** | 20817 | 27336 | 367 | 20403 | 27193 | 21232 | 21219 | 21154 | 20800 | 20809 | 21047 | 20817 | 0 |

**Table S2 - Number of core genome SNPs.**

The number of core genome SNPs on the first isolates from patients P1-P4 and P6-P14 were assessed using snp-dists 0.8.2 on the core genome alignment across all isolates. Maximum and minimum SNP distances are marked in bold and highlighted in light orange. The numbers in superscript and the colours represent the different MLST: 1, 5, 8, 22, 30, 59, 5050, 10931.

**Table S3**

|  |  |  | **Isolate** | | | | |  |
| --- | --- | --- | --- | --- | --- | --- | --- | --- |
| **Patient** | **Protein** | **Position** | **1** | **2** | **3** | **4** | **5** | **Aminoacid substitution** |
| **P1** | N-acetyl-gamma-glutamyl-phosphate reductase | 89 | T | T | C |  |  | I>T |
|  | Ribosome biogenesis GTPase A | 53 | G | G | A |  |  | S>N |
|  | 50S ribosomal protein L4 | 380 | A | G | G | G |  | D>G |
| **P3** | **Putative surface protein (LPXTG-anchored surface protein SasG)** | **Different gene length** | **3348 bp** | **2580 bp** | **567 bp** | **567 bp** |  |  |
|  | Cystathionine gamma-synthase/O-acetylhomoserine (thiol)-lyase | 676 | A | G | G | G |  | T >A |
|  | N5-carboxyaminoimidazole ribonucleotide synthase | 95 | G | A | A | A |  | G>D |
| **P4** | Phosphoenolpyruvate-protein phosphotransferase | 361 | T | G | G | G | G | Y>D |
|  | hypothetical | 643 | G | A | A | A | A | G>S |
|  | **Collagen adhesin** | **Different gene length** | **2290 bp** | **869 bp** |  |  |  |  |
|  | hypothetical | 251 | G | G | G | A | G | G>D |
|  | hypothetical | 257 | C | C | C | C | G | T>R |
|  | 6-phospho-beta-galactosidase | 515 | G | G | G | A | G | G>D |
| **P6** | hypothetical | 168 | T | T | A |  |  | S>R |
|  | Serine/threonine phosphatase stp | 638 | G | G | A |  |  | G>D |
|  | Ribonuclease Y | 1436 | G | A | A |  |  | G>D |
|  | Serine--tRNA ligase | 1240 | A | G | G |  |  | I>V |
|  | Serine-aspartate repeat-containing protein D | 3843 | T | T | A |  |  | D>E |
|  | Galactokinase | 529 | A | A | G |  |  | I>V |
|  | hypothetical | 865 | G | G | A |  |  | V>I |
|  | Lysostaphin resistance protein A | 49 | G | G | A |  |  | V>I |
|  | Fatty acid resistance protein FarB | 640 | A | A | G |  |  | M>L |
| **P7** | Ribonuclease J 1 | 1217 | G | G | A | G |  | C>Y |
|  | Putative heme-dependent peroxidase | 287 | A | A | G | A |  | D>G |
|  | Phosphatidylglycerol lysyltransferase | 2476 | C | C | T | C |  | L>F |
|  | Response regulator protein GraR | 238 | C | C | T | C |  | R>C |
|  | Diacylglycerol kinase | 215 | A | A | G | A |  | N>S |
| **P9** | YtpR family tRNA-binding protein | 472 | C | C | T |  |  | R>C |
|  | DNA-directed RNA polymerase subunit alpha | 565 | A | A | G |  |  | S>G |
|  | Sensor protein SrrB | 1264 | C | T | C |  |  | R>C |
|  | Spermidine/putrescine import ATP-binding protein PotA | 152 | C | C | T |  |  | A>V |
|  | Phage major capsid protein | 337 | A | A | G |  |  | K>E |
|  | Transcription-repair-coupling factor | 3190 | T | C | C |  |  | C>R |
|  | ATP-binding protein | 2024 | C | C | T |  |  | T>I |
| **P14** | DM13 domain-containing protein | 154 | C | A | C | C |  | L>I |
|  | Phosphatase YwpJ | 787 | G | A | G | G |  | D>N |
|  | DUF443 family protein | 26 | A | A | A | G |  | N>S |

**Table S3 – Strains in-patient evolution.**

Isolates sampled from patients at different time points were sequenced. This table summarizes the single nucleotide polymorphisms (SNPs), or gene length variations present between strains, as compared to the first isolate. SNPs are marked in red.
